# Sclerema and Œdema Neonatorum

**Published:** 1890-04-26

**Authors:** 


### The Practitioner's Mirror and Retrospect.

[*The Editor will be glad to receive offers of co-operation and contributions from members of the profession. There is no doubt that much valuable material full of interest and instruction is at present lost to science. This is largely so in the case of* (1) *the younger and less-known members of the hospital staff* (2) *those connected with cottage hospitals, and* (3) *the great body of medical practitioners not attached to any institution. The co-operation of all is cordially invited to enable the Editor to make* The Hospital *of the utmost possible use and value to the profession. Specialists willing to review books on their special subjects are invited to communicate this fact at once. All letters should be addressed to* The Editor, The Lodge, Porchester Square, London, W.]

### MEDICINE.

### SCLEREMA AND ŒDEMA NEONATORUM.

Remarks on Sclerema and Œdema neonatorum, by Dr. J. W. Ballantyne, appear in the *British Medical Journal.* These are affections of the new-born infant, regarding which some confusion exists. Sclerema may be defined as a rare disease, characterized by induration of the subcutaneous tissue. Œdema neonatorum may be defined as a disease characterized by serous infiltration. But it is due in most instances to infantile cardiac, renal, or pulmonary disease, and is, therefore, rather a symptom than a distinct pathological entity. The microscopical examination of the kidneys in an œdema case revealed the cause of the subcutaneous serous infiltration, the organs being enormously engorged with swelling of the cells of the tubules, and cell infiltration of the Malpighian bodies. In the scleremic infant, Dr. Ballantyne opines the primary pathological factor to be "a trophic lesion" of the nervous system. In both sclerema and œdema neonatorum the infants are weakly, often prematurely born, and in both diseases the temperature is below normal. In sclerema the condition of the skin is found most on the back, shoulders, and thighs; while in œdema, the lower part of the abdomen, the back, and legs are chiefly affected. In sclerema the skin is firm and tense, cannot be raised into folds, and does not pit on pressure. In œdema the skin is soft and baggy, can be pinched up between the fingers, and pits readily on pressure. In both diseases the author states the infant should be placed in an incubator. Friction with a stimulating liniment is also indicated. In œdema, when there is suppression of urine, digitalis fomentations should be used to the loins, and diuretics internally.

